# Genome-wide selection signatures in Ramlıç sheep identified by complementary ROH and iHS analyses

**DOI:** 10.3389/fvets.2026.2009151

**Published:** 2026-10-13

**Authors:** Orhan Eser, Mustafa Tekerli, Mustafa Demirtaş, Samet Çinkaya, Eda Demirtaş, Metin Erdoğan, Koray Çelikeloğlu

**Affiliations:** 1 Department of Animal Science, Faculty of Veterinary Medicine, Afyon Kocatepe University, Afyonkarahisar, Türkiye; 2 Department of Medical Biology and Genetics, Faculty of Veterinary Medicine, Afyon Kocatepe University, Afyonkarahisar, Türkiye

**Keywords:** autozygosity, genomic inbreeding, integrated haplotype score, positive selection, runs of homozygosity, sheep genomics

## Abstract

**Introduction:**

Ramlıç is a composite sheep breed developed in Türkiye through crossbreeding between Rambouillet and Dağlıç sheep followed by selection for economically important traits. This study aimed to characterize genomic autozygosity and identify putative selection signatures in Ramlıç sheep by integrating runs of homozygosity (ROH) and integrated haplotype score (iHS) analyses.

**Methods:**

A total of 96 animals were genotyped using the Illumina OvineSNP50K BeadChip, and 44,171 autosomal SNPs were retained after quality control.

**Results:**

Overall, 1,956 ROH segments were identified, with an average of 20.38 ROH per individual and a mean ROH-based genomic inbreeding coefficient (F_ROH_ of 4.57%). Short-to-intermediate ROH predominated, with segments of 2–<8 Mb accounting for approximately 78% of all detected ROH. Genome-wide analysis identified 66 ROH islands, with the highest maximum ROH incidence observed on OAR6. Haplotype-based analysis identified 48 candidate 500-kb windows containing at least three SNPs exceeding the empirical top 1% |iHS| threshold. Integrating both approaches revealed 15 concordant ROH–iHS regions on OAR1, OAR2, OAR6, and OAR11. The most prominent concordance was observed on OAR6 at 36.5–38.0 Mb, where the ROH island with the highest maximum incidence coincided with a cluster of extreme |iHS| signals and encompassed candidate genes including *ABCG2, SPP1, MEPE, LAP3,* and *NCAPG*, which have previously been associated with economically relevant traits in sheep.

**Discussion:**

These findings provide new insights into genomic autozygosity and the potential selection history of Ramlıç sheep and highlight candidate regions that warrant further investigation in larger populations using higher-resolution genomic data and functional validation.

## 1 Introduction

Sheep (*Ovis aries*) are among the most important livestock species worldwide because of their contribution to the production of meat, milk, wool, and other valuable products (1). Archeological and genomic evidence indicates that sheep were domesticated approximately 10,500 years ago in the Fertile Crescent and subsequently dispersed across diverse geographical and environmental conditions (2). During this process, sheep populations were exposed to both natural selection imposed by local environmental conditions and artificial selection for human-preferred traits. Early selection primarily targeted traits such as behavior, body size, pigmentation, and adaptation, whereas subsequent breeding objectives increasingly focused on specialized production traits, including meat, wool, milk, and skins/pelts (3). The cumulative effects of long-term selection and adaptation to diverse environments have contributed to the extensive phenotypic and genomic diversity observed among contemporary sheep breeds (4, 5). These processes can alter allele and haplotype frequencies, generating characteristic genomic patterns referred to as selection signatures (3). In sheep, such signatures have been identified in genomic regions associated with production traits, morphology, pigmentation, climatic adaptation, and disease or parasite resistance (2, 6).

Composite breeds are particularly informative for investigating breed formation and selection history at the genomic level because they arise through the combination of distinct genetic backgrounds by crossbreeding followed by selection (7). Ramlıç, a composite sheep breed developed in Türkiye, was established to combine the environmental hardiness of the indigenous Dağlıç with the superior meat and wool characteristics of Rambouillet sheep. Through crossbreeding between Rambouillet and Dağlıç sheep followed by selection, Ramlıç was developed with an approximately 65–70% Rambouillet and 30–35% Dağlıç genetic background (8). Ramlıç sheep are predominantly raised in the elevated inland areas of western Anatolia, which are characterized by a continental climate with cold winters and relatively dry summers. Subsequent studies indicate that breeding and selection efforts in the Ramlıç population have addressed economically important traits, including growth and body weight, fleece characteristics, prolificacy, and meat and carcass traits (9–11). This history of crossbreeding and selection provides a strong rationale for investigating the genomic footprints of selection in the Ramlıç population.

Runs of homozygosity (ROH) are continuous homozygous chromosomal segments resulting from the inheritance of identical haplotypes from both parents. The number, length, and genomic distribution of ROH provide information on individual autozygosity as well as the demographic and breeding history of populations (12). Long ROH segments are generally associated with more recent common ancestry and inbreeding, whereas shorter segments tend to reflect more ancient demographic processes. The ROH-based genomic inbreeding coefficient (F_ROH_), defined as the proportion of the autosomal genome covered by ROH, can therefore be used to quantify genomic inbreeding (12, 13). Moreover, because the genomic distribution of ROH can provide information on regions potentially affected by selection, ROH analysis has also been used to investigate putative selection signatures (13, 14).

The integrated haplotype score (iHS) is a haplotype-based approach derived from extended haplotype homozygosity (EHH) that detects signals of positive selection by comparing the decay of haplotype homozygosity associated with alternative alleles at a locus. The method is particularly effective for detecting recent positive selection when the selected allele has increased in frequency but has not yet reached fixation (15). Because no single selection-signature method is expected to capture all selective processes with equal sensitivity, approaches based on different genomic features can provide complementary information when applied jointly (3). In this context, ROH characterize the genomic distribution of homozygous segments, whereas iHS targets extended haplotype structures associated with recent positive selection; genomic regions supported by multiple approaches can therefore be prioritized as candidate selection signals. Genome-wide studies in sheep have demonstrated the utility of combining ROH, iHS, and other selection statistics to identify breed-specific and shared selection signals and candidate genomic regions associated with production, reproduction, immunity, and adaptation (3, 16–18).

Despite the economic and genetic importance of Ramlıç sheep, previous studies have largely focused on production and phenotypic traits, whereas their genome-wide autozygosity patterns and selection history remain comparatively underexplored. In particular, the extent to which ROH-based selection signals overlap with haplotype-based evidence of recent positive selection in Ramlıç sheep has not yet been established. We hypothesized that the breeding and selection history of the Ramlıç population has left detectable genomic footprints and that the joint evaluation of ROH and iHS would identify genomic regions potentially influenced by selection and supported by both approaches. Accordingly, this study aimed to characterize genome-wide patterns of homozygosity and genomic inbreeding in Ramlıç sheep and to identify putative selection signatures using complementary ROH- and iHS-based approaches. To this end, ROH characteristics and F_ROH_ were evaluated, ROH islands and iHS-based selection signals were identified, and genomic regions supported by both approaches were prioritized for candidate gene identification and biological interpretation.

## 2 Materials and methods

### 2.1 Ethical statement

This study was based on microarray genotype data previously generated from animals genotyped within a research project supported by the Scientific Research Projects Coordination Unit of Afyon Kocatepe University (Project No. 22. SAĞ. BİL.13). The collection of biological samples and genotyping of these animals were conducted under the approval of the Animal Experiments Local Ethics Committee of Afyon Kocatepe University (AKUHADYEK), Türkiye (Approval No. 49533702/58; Decision No. 50/22; approved on 2 June 2022). The present study involved secondary analysis of the previously generated genotype data and did not involve additional animal sampling or experimental procedures.

### 2.2 Animals, DNA extraction, and genotyping

The study population consisted of 96 Ramlıç sheep raised at the Education, Research and Application Farm of the Faculty of Veterinary Medicine, Afyon Kocatepe University, during the 2022–2023 breeding and lambing season. Blood samples were collected from the jugular vein into tubes containing tripotassium ethylenediaminetetraacetic acid (K₃EDTA) and stored at −20 °C until DNA extraction. Genomic DNA was extracted using the Quick-DNA™ Miniprep Kit (Zymo Research, USA). DNA concentration was measured using the Qubit™ 1X dsDNA Broad Range Assay Kit with a Qubit 2.0 Fluorometer (Thermo Fisher Scientific, USA), and DNA purity was assessed based on A260/280 and A260/230 absorbance ratios using a Multiskan GO Microplate Spectrophotometer (Thermo Fisher Scientific, USA). DNA samples from all 96 animals were genotyped using the Illumina OvineSNP50K BeadChip on an Illumina iScan platform at Refgen Biotechnology Laboratories. Genotyping data were processed using GenomeStudio 2.0 (Illumina Inc., San Diego, CA, USA), yielding an initial dataset of 64,734 SNPs.

### 2.3 Genotype quality control

Of the initial 64,734 SNPs, 1,955 non-autosomal markers were excluded, leaving 62,779 autosomal SNPs. Missing genotypes were imputed using EasyIMPUTE, which implements a k-nearest neighbor (kNN) algorithm (19). Following imputation, SNPs with a minor allele frequency (MAF) < 0.05 or deviating from Hardy–Weinberg equilibrium $\left(P<1\times 10^{-4}\right)$ were excluded (20). After quality control, 44,171 SNPs distributed across the 26 ovine autosomes were retained for subsequent genomic analyses.

### 2.4 Runs of homozygosity analysis

Runs of homozygosity (ROH) were identified from the quality-controlled autosomal SNP dataset using the sliding-window method implemented in the detectRUNS package in R (21). The minimum number of SNPs required to define an ROH was calculated using the approach described by Purfield et al. (12) as:


$$l=\frac{\ln\frac{\alpha }{n_{s}.n_{i}}}{\ln(1-\bar{H})}$$

Where $n_{s}$ is the number of SNPs per individual, $n_{i}$ is the number of individuals, α is the expected proportion of false-positive ROH, and $\bar{H}$ is the mean SNP heterozygosity. The minimum number of SNPs was calculated in R (v4.3.3) using a custom script implementing the approach described by Purfield et al. (12). Based on 44,171 autosomal SNPs, 96 animals, a mean heterozygosity of 0.39187, and α=0.05, the estimated minimum was 36.70 and was rounded up to 37 SNPs.

ROH were detected using a 37-SNP sliding window and a window threshold of 0.05, with a minimum of 37 SNPs per ROH. A maximum of one heterozygous and two missing genotypes was allowed per window. The minimum ROH length was set to 1 Mb, the maximum gap between consecutive SNPs to 1 Mb, and the minimum marker density to one SNP per 100 kb (12, 13, 17).

For each individual, the number of ROH (N_ROH_), total ROH length (L_ROH_) and mean ROH length were calculated. ROH were classified according to physical length as 1–<2, 2–<4, 4–<8, 8–<16, and ≥16 Mb (22). The ROH-based genomic inbreeding coefficient (F_ROH_) was calculated following McQuillan et al. (23) as:


$$F_{\mathrm{ROH}}=\frac{\sum L_{\mathrm{ROH}}}{L_{\text{AUTO}}}$$

Where $\sum L_{\mathrm{ROH}}$ is the cumulative length of ROH detected in an individual and $L_{\text{AUTO}}$ is the total autosomal genome length covered by the SNP panel, calculated as the sum of the physical distances between the first and last retained SNP on each of the 26 autosomes. Class-specific $F_{\mathrm{ROH}}$ values were also calculated for each ROH length category.

To identify ROH islands, the incidence of each SNP within ROH was calculated as the proportion of animals in which the SNP was encompassed by an ROH. SNPs occurring within ROH in at least 10% of the population were retained as high-frequency ROH loci, following the threshold applied by Karabaş and Yılmaz (17). In the present population of 96 animals, this criterion corresponded to a minimum of 10 animals (10/96 = 10.42%). Consecutive qualifying SNPs on the same chromosome, based on their original order in the SNP map, were grouped into ROH islands. For each island, chromosome, start and end positions, physical length, number of SNPs, maximum ROH incidence, and the SNP with the highest ROH frequency were determined.

### 2.5 Integrated haplotype score analysis

The integrated haplotype score (iHS) approach was used to identify genomic signatures of recent positive selection based on extended haplotype homozygosity (EHH) (15). Following genotype quality control, haplotypes were phased using Beagle v5.1 with default settings, and iHS values were calculated chromosome-wise from the quality-controlled dataset comprising 44,171 autosomal SNPs using selscan v3.0.1 (24) with the --ihs and --pmap options. Physical marker positions were used for EHH-based calculations.

Because reliable ancestral and derived allele information was unavailable, allelic states were not polarized. Raw iHS values generated by selscan were subsequently standardized in R using a custom script within allele-frequency bins of width 0.01 to account for the frequency-dependent distribution of the statistic (15, 24):


$$\mathrm{iHS}=\frac{\ln(\frac{\mathrm{iH}H_{1}}{\mathrm{iH}H_{0}})-E_{P}[\ln(\frac{\mathrm{iH}H_{1}}{\mathrm{iH}H_{0}})]}{SD_{P}[\ln(\frac{\mathrm{iH}H_{1}}{\mathrm{iH}H_{0}})]}$$

Where $\mathrm{iH}H_{0}$ and $\mathrm{iH}H_{1}$ represent the integrated haplotype homozygosity (iHH) values associated with the two allelic states coded as 0 and 1, respectively, and $E_{P}$ and $SD_{P}$ represent the expected value and standard deviation of the log-transformed iHH ratio within allele-frequency bin p, respectively. Because ancestral and derived allele states were not assigned, the sign of the standardized iHS statistic was not interpreted, and subsequent analyses were based on absolute iHS values ( ∣iHS∣ ) (25, 26).

Extreme iHS signals were identified from the empirical genome-wide distribution of ∣iHS∣ . SNPs within the upper 1% of the distribution were considered extreme (27, 28), corresponding to a 99th-percentile threshold of ∣iHS∣≥2.7232 in the present dataset.

To identify regional selection signatures, the autosomal genome was divided into non-overlapping 500-kb windows. Windows containing at least three SNPs exceeding the genome-wide top 1% ∣iHS∣ threshold were considered candidate regions showing signatures of recent positive selection (28, 29).

### 2.6 Overlap of ROH Islands and iHS candidate regions

ROH islands and iHS candidate regions were compared based on their chromosomal positions and physical coordinates to identify genomic regions showing concordant signals between the two approaches. An overlap was defined when an ROH island and an iHS candidate region occurred on the same chromosome and shared at least one base pair (18, 29). All overlap analyses and related calculations were performed in R using custom scripts. For each overlapping pair, the shared genomic interval and its physical length were determined. Overlapping regions were retained for subsequent candidate gene annotation.

### 2.7 Candidate gene annotation

Candidate genes within the ROH–iHS overlapping regions were identified based on the *Ovis aries* Oar_v3.1 genome assembly using the NCBI Genome Data Viewer. Genes whose annotated genomic coordinates overlapped these regions were retained for subsequent biological interpretation.

## 3 Results

### 3.1 Distribution of runs of homozygosity and genomic inbreeding

Runs of homozygosity (ROH) showed considerable inter-individual variation in the Ramlıç population. Across the 96 genotyped animals, the mean number of ROH per individual (N_ROH_) was 20.38, ranging from 5 to 53, whereas the mean cumulative ROH length was 111.38 Mb, with individual values ranging from 13.08 to 570.92 Mb. The average of the individual mean ROH lengths was 4.82 Mb, with a median of 4.08 Mb (Table 1). The genomic inbreeding coefficient estimated from ROH (F_ROH_) averaged 4.57%, with a median of 3.31% and a wide individual range from 0.54 to 23.41% (Table 1). The higher mean than median F_ROH_, together with the broad individual range, reflected the presence of a limited number of animals with comparatively high ROH-based inbreeding levels. A total of 1,956 ROH segments were identified across the population. The ROH length distribution was dominated by short and intermediate segments. ROH of 2–<4 Mb constituted the largest proportion of all detected segments (52.20%), followed by ROH of 4–<8 Mb (26.23%). Together, these two classes accounted for approximately 78% of all ROH segments, whereas progressively fewer segments were observed in the longer ROH categories (Table 2).

**Table 1:** Population-level summary of runs of homozygosity (ROH) and genomic inbreeding in Ramlıç sheep.

Parameter	Mean	Median	Minimum	Maximum
Number of ROH per individual (N_ROH_)	20.38	18.00	5	53
Total ROH length (Mb)	111.38	80.65	13.08	570.92
Mean ROH length (Mb)	4.82	4.08	2.62	13.28
F_ROH_ (%)	4.57	3.31	0.54	23.41

ROH statistics were calculated for 96 genotyped Ramlıç sheep. N_ROH_ represents the number of ROH segments identified per individual; total ROH length is the cumulative length of all ROH segments per individual; mean ROH length is the average length of ROH segments per individual. F_ROH_ was calculated as the proportion of the autosomal genome covered by ROH and is presented as a percentage. ROH, runs of homozygosity; F_ROH_, genomic inbreeding coefficient based on ROH.

**Table 2:** Distribution of runs of homozygosity (ROH) according to length classes in Ramlıç sheep.

ROH length class	Number of ROH	Percentage (%)
1–<2 Mb	126	6.44
2–<4 Mb	1,021	52.20
4–<8 Mb	513	26.23
8–<16 Mb	196	10.02
≥16 Mb	100	5.11

ROH segments identified across the 96 genotyped Ramlıç sheep were classified according to their physical length into five categories: 1–<2 Mb, 2–<4 Mb, 4–<8 Mb, 8–<16 Mb, and ≥16 Mb. Percentages represent the proportion of ROH segments in each length class relative to the total number of detected ROH segments. ROH, runs of homozygosity; Mb, megabase.

### 3.2 Genome-wide distribution of ROH Islands

The genome-wide distribution of SNP incidence within ROH revealed substantial heterogeneity among autosomes, with several localized regions showing higher SNP incidence within ROH across individuals (Figure 1). SNPs occurring within an ROH in at least 10 of the 96 animals (≥10.42%) were used to delineate ROH islands. Based on this criterion, 66 ROH islands were identified across the autosomal genome (Supplementary Table S1), with the 20 regions showing the highest maximum ROH frequencies summarized in Table 3. The ROH island with the highest maximum ROH frequency was observed on OAR6, spanning 30.332–40.001 Mb (9.669 Mb) and containing 159 SNPs. The peak SNP, OAR6_40780034.1 at 36.573 Mb, occurred within an ROH in 25 of the 96 animals, corresponding to the highest observed ROH frequency of 26.04%. A second extended ROH island was detected on the same chromosome between 43.754 and 49.733 Mb, spanning 5.979 Mb and 114 SNPs, with a maximum ROH frequency of 19.79%. Other ROH islands with relatively high maximum frequencies included OAR2 at 21.690–24.222 Mb, with a maximum frequency of 16.67%, and regions on OAR3 (28.150–30.986 Mb) and OAR19 (20.155–25.753 Mb), both reaching a maximum frequency of 15.62%. Additional ROH islands were identified on OAR1, OAR2, OAR3, OAR7, OAR9, OAR11, OAR15–OAR18, and OAR24, generally exhibiting maximum ROH frequencies between 11.46 and 13.54% (Table 3).

**Figure 1:**
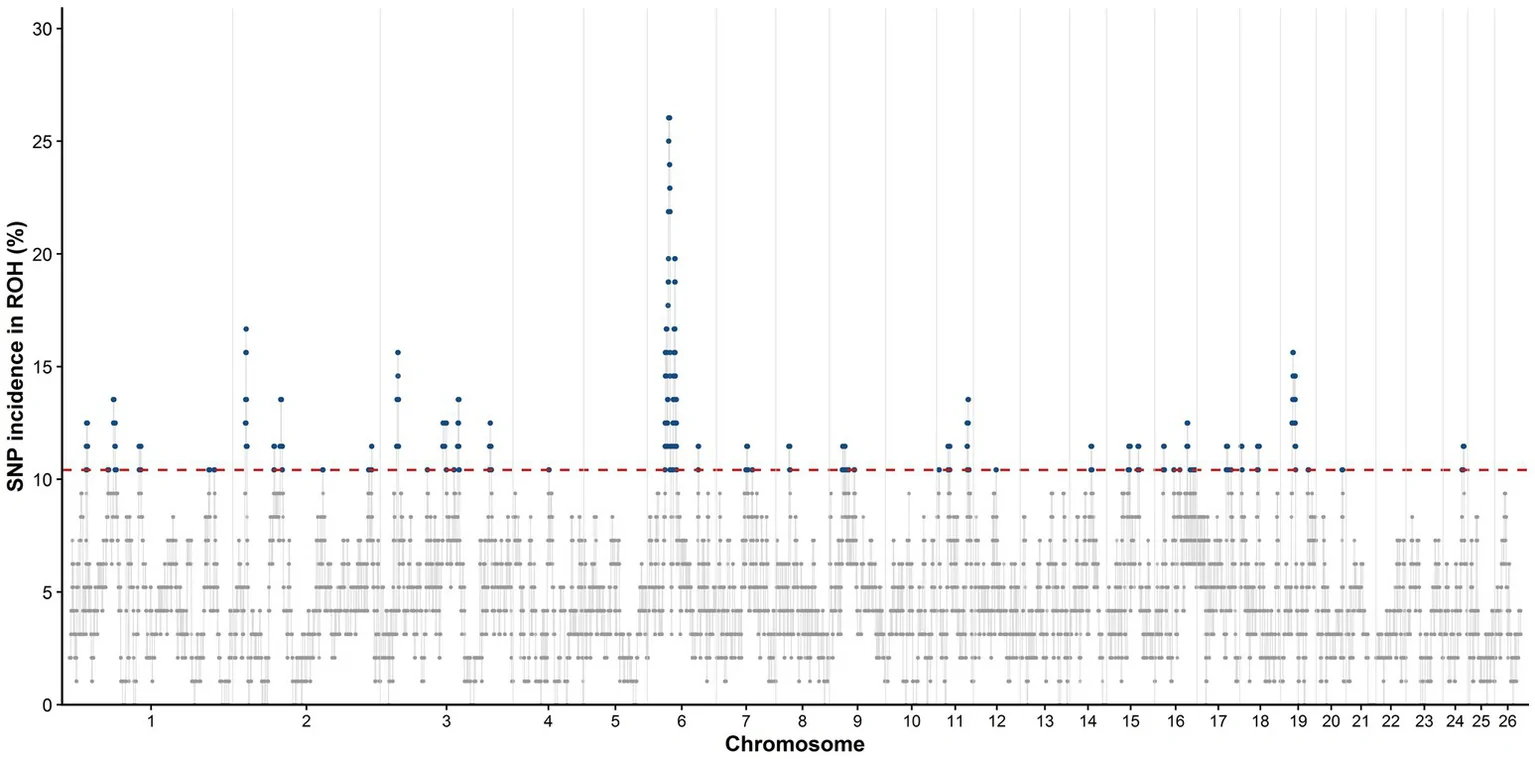
Genome-wide distribution of SNP incidence within runs of homozygosity (ROH) in Ramlıç sheep.

**Table 3:** Major runs of homozygosity (ROH) islands identified in Ramlıç sheep.

OAR	Start (Mb)	End (Mb)	Length (Mb)	SNPs	Max. ROH (n/96)	Max. frequency (%)	Peak SNP	Peak position (Mb)
6	30.332	40.001	9.669	159	25	26.04	OAR6_40780034.1	36.573
6	43.754	49.733	5.979	114	19	19.79	OAR6_51969295.1	46.891
2	21.690	24.222	2.532	55	16	16.67	s72217.1	22.763
19	20.155	25.753	5.598	94	15	15.62	OAR19_22671203.1	21.576
3	28.150	30.986	2.835	57	15	15.62	OAR3_32039232.1	29.886
1	74.387	78.138	3.752	84	13	13.54	OAR1_79590680.1	74.387
2	80.033	83.820	3.787	79	13	13.54	OAR2_86146743.1	80.984
3	130.889	133.443	2.553	48	13	13.54	OAR3_140679608.1	131.721
11	51.552	53.676	2.124	37	13	13.54	OAR11_56856134.1	53.128
3	105.894	111.747	5.853	105	12	12.50	OAR3_112822823.1	106.053
3	184.444	187.634	3.190	58	12	12.50	OAR3_200054679.1	185.804
1	28.392	30.493	2.101	40	12	12.50	s32583.1	29.284
16	54.870	56.088	1.218	25	12	12.50	OAR16_60075688.1	55.120
9	21.946	24.527	2.581	60	11	11.46	OAR9_23529045.1	22.624
17	49.864	52.959	3.096	50	11	11.46	s10425.1	50.179
24	32.268	35.559	3.291	47	11	11.46	s03477.1	34.461
7	51.221	53.207	1.986	45	11	11.46	OAR7_56597763.1	51.221
18	30.478	32.888	2.410	44	11	11.46	s19976.1	30.478
15	52.773	55.204	2.431	43	11	11.46	s04508.1	52.945
16	14.767	16.868	2.101	43	11	11.46	OAR16_17147237.1	15.515

The table presents the 20 major ROH islands ranked according to their maximum ROH frequency. ROH islands were defined from consecutive SNPs showing elevated occurrence within ROH across the 96 genotyped Ramlıç sheep. Max. ROH (n/96) indicates the highest number of individuals in which a SNP within the island occurred in an ROH, and maximum frequency (%) represents the corresponding proportion of the 96 animals. Peak SNP denotes the SNP with the highest ROH frequency within each island. The complete set of 66 identified ROH islands can be provided as Supplementary material. OAR, *Ovis aries* autosome; ROH, runs of homozygosity; SNP, single nucleotide polymorphism; Mb, megabase.

### 3.3 Genome-wide iHS signatures of selection

The genome-wide distribution of standardized absolute iHS values revealed multiple loci with elevated haplotype-based selection signals across the 26 ovine autosomes (Figure 2). Using the empirical top 1% threshold ( ∣iHS≥2.7232∣ ), candidate SNPs were distributed across multiple chromosomes, with variation in the magnitude and local concentration of the signals across the genome. To identify genomic regions characterized by clusters of extreme iHS values rather than isolated SNP signals, the genome was further evaluated using non-overlapping 500-kb windows. Windows containing at least three SNPs exceeding the empirical top 1% |iHS| threshold were defined as candidate regions. This procedure identified 48 candidate windows distributed across 20 autosomes (Figure 3; Supplementary Table S2). Among these, 26 windows across 13 autosomes contained at least 50% of their SNPs above the top 1% threshold and are summarized in Table 4. Several of the windows with the highest proportions of top 1% SNPs were observed on OAR6. All SNPs within the 32.0–32.5 Mb (8/8), 32.5–33.0 Mb (5/5), 36.5–37.0 Mb (6/6), and 37.0–37.5 Mb (7/7) windows exceeded the empirical threshold. The 36.5–37.0 Mb interval reached a maximum ∣iHS∣ of 6.011, followed by the adjacent 37.0–37.5 Mb interval (maximum ∣iHS∣ = 5.697) and the 32.5–33.0 Mb interval (maximum |iHS| = 5.492). The neighboring 37.5–38.0 Mb window contained eight of nine SNPs (88.89%) exceeding the top 1% threshold. On OAR7, eight of 10 SNPs in the 21.0–21.5 Mb window exceeded the threshold, with a maximum |iHS| of 5.498. All SNPs also exceeded the threshold in the OAR9 50.0–50.5 Mb window (7/7 SNPs) and the OAR24 10.5–11.0 Mb window (6/6 SNPs). Additional candidate regions summarized in Table 4 were located on OAR2, OAR5, OAR9, OAR11, OAR12, OAR13, OAR21, OAR23, OAR25, and OAR26. OAR11 contained several distinct candidate regions. The 22.0–22.5 and 52.5–53.0 Mb windows each contained seven top 1% SNPs out of nine (77.78%), with maximum |iHS| values of 4.099 and 4.974, respectively, while the 56.0–56.5 Mb interval contained six of seven SNPs (85.71%) exceeding the top 1% threshold.

**Figure 2:**
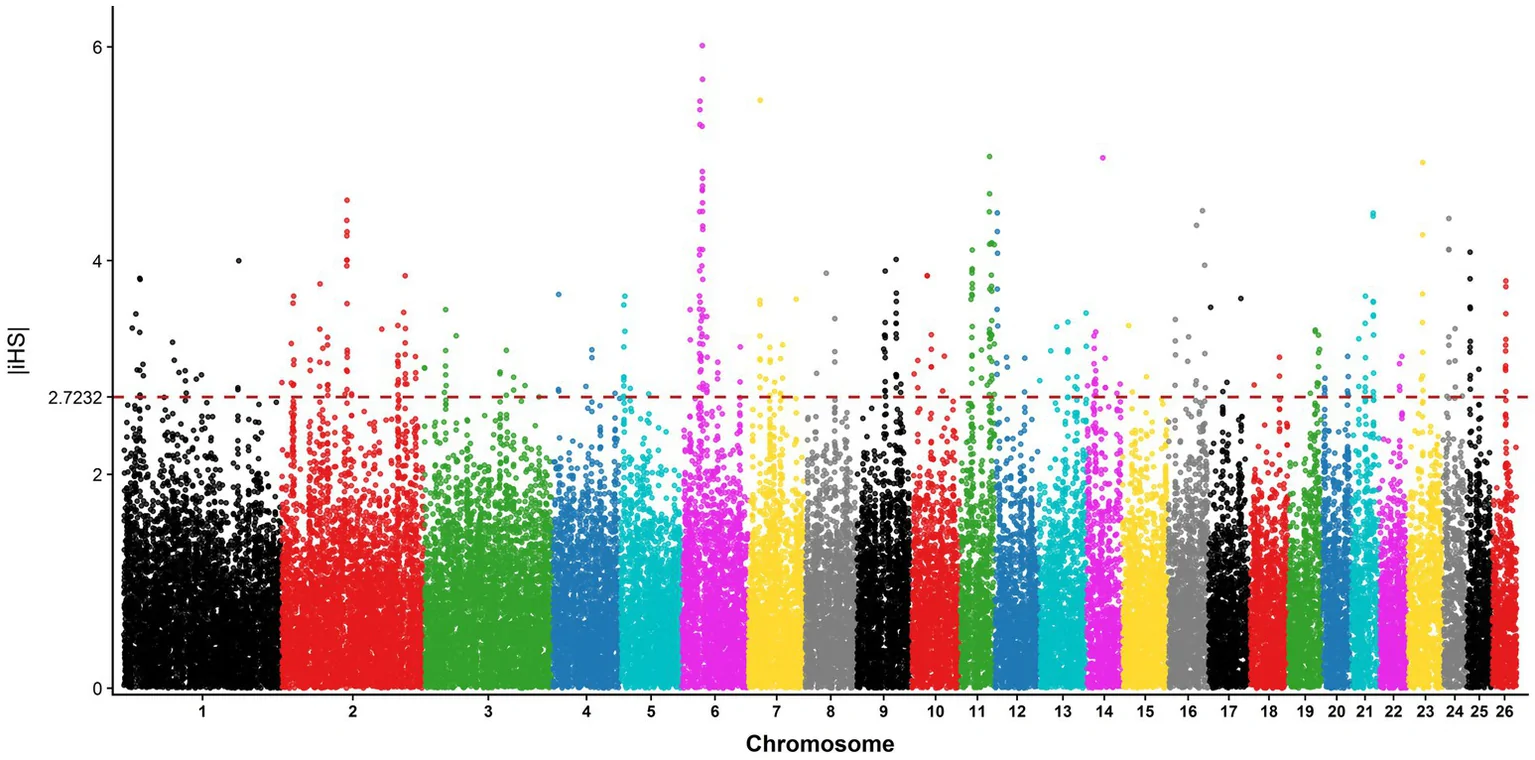
Genome-wide distribution of standardized |iHS| values in Ramlıç sheep.

**Figure 3:**
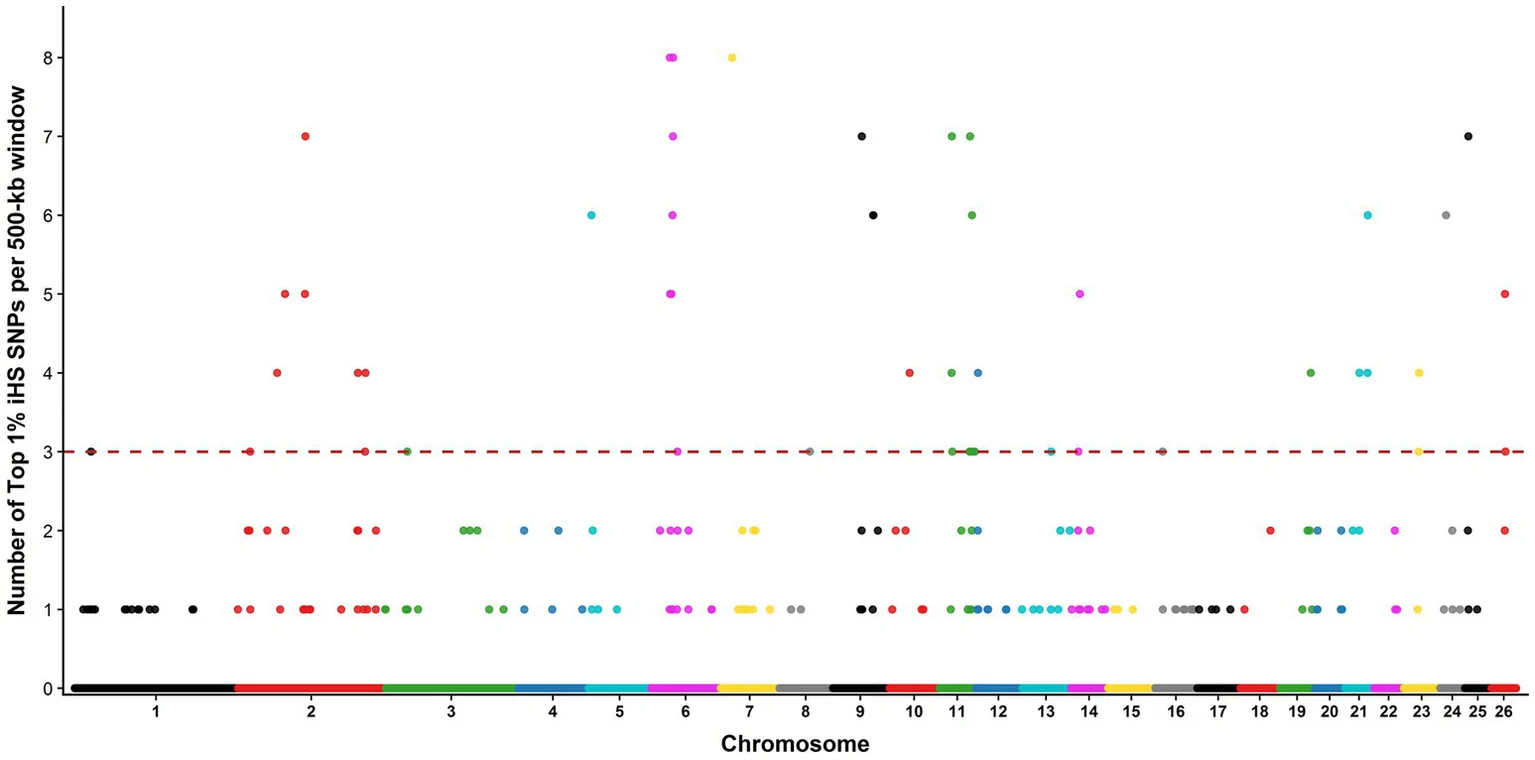
Genome-wide distribution of top 1% iHS SNPs in 500-kb windows in Ramlıç sheep.

**Table 4:** Major genomic regions showing strong iHS-based signatures of selection in Ramlıç sheep.

OAR	Genomic region (Mb)	SNPs in window	Top 1% SNPs	Top 1% (%)	Max |iHS|
2	114.0–114.5	6	5	83.33	4.376
2	114.5–115.0	10	7	70.00	4.564
2	215.5–216.0	8	4	50.00	3.859
5	6.0–6.5	9	6	66.67	3.586
6	32.0–32.5	8	8	100.00	4.458
6	32.5–33.0	5	5	100.00	5.492
6	36.5–37.0	6	6	100.00	6.011
6	37.0–37.5	7	7	100.00	5.697
6	37.5–38.0	9	8	88.89	4.324
6	44.5–45.0	6	3	50.00	3.476
7	21.0–21.5	10	8	80.00	5.498
9	50.0–50.5	7	7	100.00	3.902
9	69.0–69.5	8	6	75.00	4.010
9	69.5–70.0	9	6	66.67	3.696
11	22.0–22.5	9	7	77.78	4.099
11	52.5–53.0	9	7	77.78	4.974
11	56.0–56.5	7	6	85.71	4.166
11	56.5–57.0	5	3	60.00	3.438
12	5.0–5.5	6	4	66.67	4.445
13	50.0–50.5	6	3	50.00	3.424
21	39.0–39.5	5	4	80.00	4.444
21	39.5–40.0	11	6	54.55	3.611
23	26.0–26.5	6	3	50.00	3.686
24	10.5–11.0	6	6	100.00	4.394
25	6.5–7.0	9	7	77.78	4.078
26	24.0–24.5	7	5	71.43	3.810

Candidate 500-kb windows were initially defined as windows containing at least three SNPs exceeding the empirical top 1% threshold (|iHS| ≥ 2.7232). The table presents the strongest candidate windows in which at least 50% of SNPs exceeded this threshold (top 1% ≥ 50%). In total, 26 windows distributed across 13 autosomes met this criterion. OAR, *Ovis aries* autosome; SNP, single nucleotide polymorphism; iHS, integrated haplotype score.

### 3.4 Concordance between ROH Islands and iHS candidate regions

Comparison of the ROH- and iHS-based selection signatures identified a subset of genomic regions showing concordant signals between the two approaches (Figure 4). Overlapping signals were detected on OAR1, OAR2, OAR6, and OAR11, resulting in 15 ROH–iHS overlapping intervals (Table 5). Most of the overlapping intervals were located on OAR6 and OAR11, which contained seven and five intervals, respectively. On OAR1, the overlap occurred at 28.500–29.000 Mb and encompassed 13 annotated genes, including *SSBP3*, *ACOT11*, *MROH7*, *TTC4*, *PARS2*, and *TTC22*. Two overlapping regions were detected on OAR2, including the 21.690–22.000 Mb interval and a second 500-kb interval at 80.500–81.000 Mb, the latter containing *TYRP1* and *LURAP1L*. OAR6 contained seven ROH–iHS overlapping intervals, the largest number observed among the four chromosomes. Three adjacent overlapping intervals extended from 36.5 to 38.0 Mb and were located within the ROH island with the highest maximum ROH frequency in the population. The 36.5–37.0 Mb interval contained seven annotated genes, including *ABCG2*, *PKD2*, *SPP1*, *MEPE*, and *IBSP*, whereas the adjacent 37.0–37.5 Mb interval contained nine annotated genes, including *LAP3*, *MED28*, *FAM184B*, *NCAPG*, and *DCAF16*. The subsequent 37.5–38.0 Mb interval contained three annotated loci. Additional OAR6 overlaps were detected at 32.0–32.5, 32.5–33.0, 34.5–35.0, and 44.5–45.0 Mb; these regions included *GRID2*, *MMRN1*, *SNCA*, *SEPSECS*, *PI4K2B*, *ZCCHC4*, and *ANAPC4*. Five overlapping intervals were identified on OAR11. The 21.549–22.000 Mb region contained 14 annotated genes, followed by a shorter 74.289-kb overlap at 22.000–22.074 Mb containing *RPH3AL*. Three contiguous 500-kb intervals between 52.0 and 53.5 Mb also showed overlapping ROH and iHS signals. These intervals contained several annotated genes, including *RBFOX3*, *C1QTNF1*, *TIMP2*, *USP36*, *CYTH1*, *SOCS3*, *BIRC5*, *TMC8*, *TMC6*, and *TNRC6C*. Overall, the concordance analysis identified 15 genomic intervals showing overlapping signals from the two complementary approaches, with the largest number of overlaps observed on OAR6.

**Figure 4:**
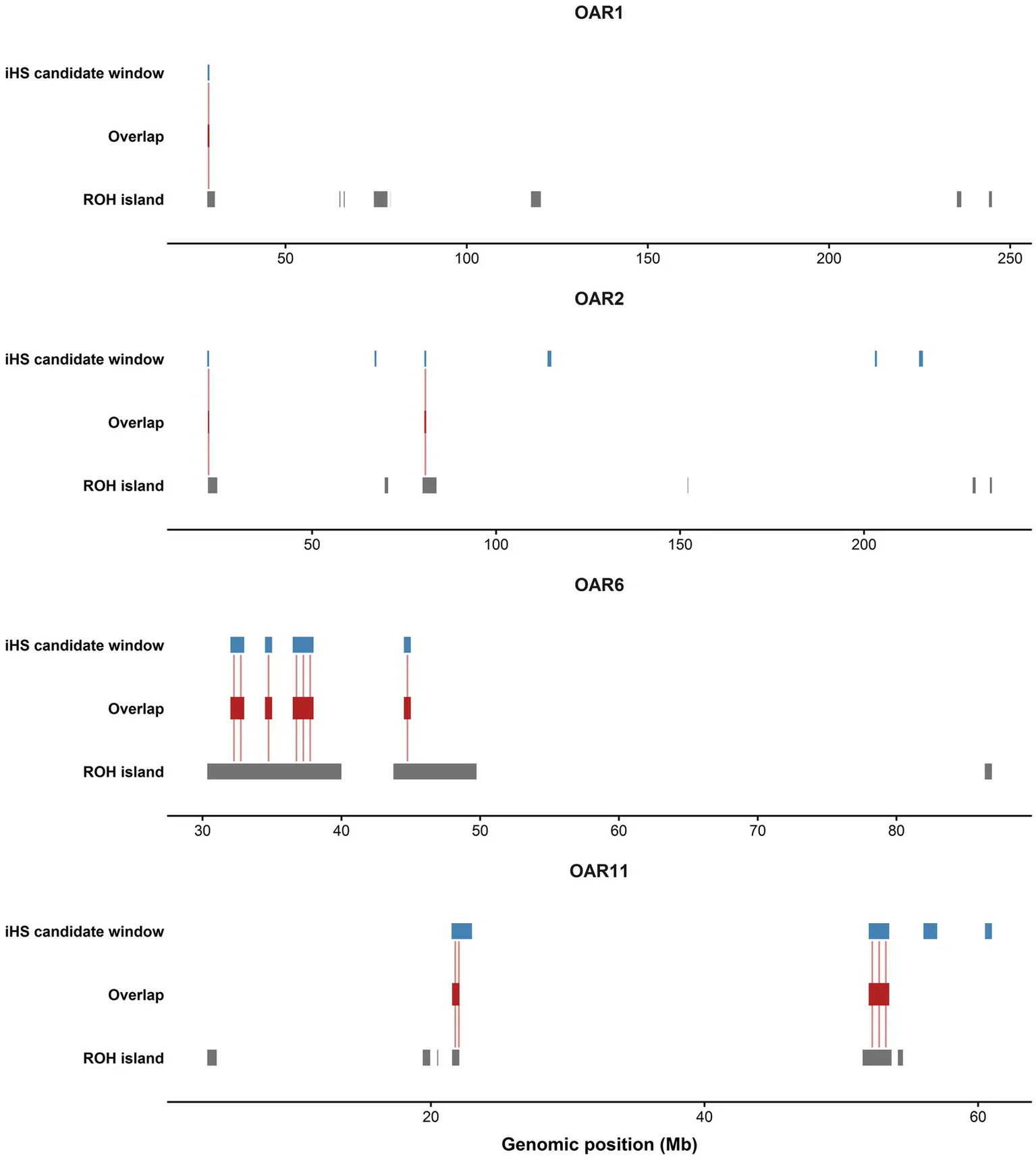
Regional concordance between ROH islands and iHS candidate regions in Ramlıç sheep.

**Table 5:** Genes located within genomic regions jointly identified by ROH and iHS analyses in Ramlıç sheep.

Chr	ROH–iHS overlapping region (bp)	Length (kb)	No. of annotated genes	Annotated genes
1	28,500,001–29,000,000	500	13	*SSBP3*, *LOC105609608*, *LOC105609607*, *LOC105611354*, *ACOT11*, *LOC101118495*, *LOC101118751*, *TRNAP-AGG*, *MROH7*, *TTC4*, *PARS2*, *C1H1orf177*, *TTC22*
2	21,690,410–22,000,000	310	3	*LOC101106149*, *LOC101106409*, *LOC101122409*
2	80,500,001–81,000,000	500	5	*TYRP1*, *LURAP1L*, *LOC105608121*, *LOC101121242*, *LOC105608132*
6	32,000,001–32,500,000	500	2	*GRID2*, *TRNAS-AGA*
6	32,500,001–33,000,000	500	1	*LOC105615445*
6	34,500,001–35,000,000	500	4	*MMRN1*, *LOC105615450*, *SNCA*, *LOC105612139*
6	36,500,001–37,000,000	500	7	*ABCG2*, *PKD2*, *SPP1*, *LOC105615454*, *MEPE*, *IBSP*, *LOC101103815*
6	37,000,001–37,500,000	500	9	*TRNAA-CGC*, *LAP3*, *MED28*, *FAM184B*, *LOC105615455*, *NCAPG*, *DCAF16*, *LOC101104320*, *LOC105615456*
6	37,500,001–38,000,000	500	3	*LOC101104320*, *LOC105608050*, *LOC105608048*
6	44,500,001–45,000,000	500	4	*SEPSECS*, *PI4K2B*, *ZCCHC4*, *ANAPC4*
11	21,548,639–22,000,000	451	14	*NXN*, *LOC105616314*, *RNMTL1*, *GLOD4*, *LOC101113864*, *GEMIN4*, *FAM57A*, *LOC105611240*, *VPS53*, *LOC105616315*, *FAM101B*, *C11H17orf97*, *RPH3AL*, *LOC105616316*
11	22,000,001–22,074,289	74	1	*RPH3AL*
11	52,000,001–52,500,000	500	9	*LOC105616411*, *RBFOX3*, *ENGASE*, *C1QTNF1*, *CANT1*, *LGALS3BP*, *CEP295NL*, *TIMP2*, *LOC105616412*
11	52,500,001–53,000,000	500	16	*TIMP2*, *USP36*, *CYTH1*, *LOC105616414*, *DNAH17*, *PGS1*, *SOCS3*, *LOC105606278*, *LOC105606280*, *LOC101113775*, *TMEM235*, *BIRC5*, *AFMID*, *TK1*, *TRNAS-GGA*, *SYNGR2*
11	53,000,001–53,500,000	500	6	*TMC8*, *TMC6*, *LOC105606334*, *TNRC6C*, *LOC105606283*, *LOC105606285*

ROH–iHS overlapping regions represent the physical intersections between ROH islands and iHS candidate regions. Gene annotation was performed using the *Ovis aries* Oar_v3.1 genome assembly.

## 4 Discussion

### 4.1 Genomic autozygosity and selection signatures

The present study provides a genome-wide characterization of autozygosity and putative selection signatures in Ramlıç sheep using complementary ROH- and iHS-based approaches. The predominance of short-to-intermediate ROH segments, together with localized regions of elevated ROH incidence and clusters of extreme |iHS| values, revealed a heterogeneous genomic landscape rather than a uniform pattern of homozygosity or haplotype differentiation across the genome. Importantly, concordance between ROH islands and iHS candidate regions identified a subset of genomic intervals supported by two approaches that capture different features of genomic variation. Such multi-method strategies can strengthen the identification and prioritization of putative selection signatures by providing complementary evidence for the same genomic regions (18, 29). Therefore, the concordant signals identified in the present study may represent particularly informative genomic footprints for understanding the breeding and selection history of the Ramlıç population.

The ROH profile of Ramlıç sheep was characterized by moderate genomic autozygosity, with a mean F_ROH_ of 4.57% and an average of 20.38 ROH per individual. These values were higher than those reported for Chinese Merino (F_ROH_ = 0.023) and Lori-Bakhtiari sheep (F_ROH_ = 0.031), but remained within the range reported across Italian sheep breeds (0.016–0.099) (30–32). The predominance of short-to-intermediate ROH, particularly segments between 2 and 8 Mb, suggests that much of the observed autozygosity reflects more distant common ancestry and historical demographic processes rather than widespread recent consanguinity, as longer ROH are generally associated with more recent common ancestry (13, 33). Nevertheless, the wide individual variation in F_ROH_ (0.0054–0.2341) indicates that some animals carry substantially greater autozygous burdens than suggested by the population mean. Overall, these findings indicate moderate population-level genomic inbreeding in Ramlıç sheep, accompanied by considerable individual heterogeneity.

The non-uniform distribution of ROH islands across the Ramlıç genome indicates that autozygosity was concentrated in specific genomic regions rather than being randomly distributed. Such recurrent homozygous regions across individuals may arise from increased homozygosity surrounding loci subjected to natural or artificial selection and have therefore been used as putative signatures of selection in livestock populations (13, 34). However, ROH patterns can also be shaped by demographic history, genetic drift, recombination and local linkage disequilibrium, and consequently ROH islands alone cannot be regarded as definitive evidence of selection (34, 35). Accordingly, combining ROH-based signals with independent selection-signature approaches can improve the prioritization of candidate regions (29, 36). This provides the rationale for interpreting the ROH islands identified in Ramlıç sheep together with the haplotype-based iHS signals.

The iHS analysis revealed clusters of extreme haplotype-based signals across multiple genomic regions in Ramlıç sheep. Because iHS is particularly sensitive to recent or ongoing positive selection in which a favored allele has increased in frequency while remaining associated with an unusually extended haplotype, the regional clustering of extreme scores is consistent with putative recent selective pressures on the Ramlıç genome (15). Similar genome-wide patterns of localized iHS signals have been reported in sheep populations with different breeding histories, supporting the utility of haplotype-based approaches for detecting recent selection in ovine genomes (3, 37, 38). Importantly, because reliable ancestral allele states were unavailable in the present study, candidate regions were defined using absolute iHS values (|iHS|). Accordingly, these signals indicate unusually extended haplotypes but do not allow inference of whether selection acted on the ancestral or derived allelic state.

### 4.2 Concordant ROH–iHS signals and their biological relevance

The concordance between ROH islands and iHS signals provides complementary evidence of putative selection in the Ramlıç genome. Although these approaches capture different genomic features, 15 regions showed concordant signals between the two methods, supporting their prioritization as candidate selection regions. Similar multi-method strategies have been used in livestock, including sheep, to increase confidence in candidate regions and reduce method-specific false-positive signals (17, 38–41). Therefore, the overlapping regions identified here represent convergent rather than definitive evidence of selection and provide a focused set of candidates for biological interpretation.

The most compelling signal in the present study was observed on OAR6, particularly across the 36.5–38.0 Mb interval, where consecutive iHS candidate windows overlapped the strongest ROH island. Notably, this interval lies within a broader genomic region repeatedly identified as a selection hotspot in independent sheep populations using different analytical approaches. Fariello et al. (42) reported a broad selection signature at 33.22–41.02 Mb encompassing *ABCG2* and *NCAPG*, while Rodríguez-Ramilo et al. (36) detected a 30–40 Mb ROH island in French dairy sheep and independently supported selection near *NCAPG*/*LCORL* using F_ST_. Similarly, the strongest ROH hotspot in Chinese Merino occurred at 35.07–38.67 Mb and included the *NCAPG*/*LCORL* locus, whereas Istrian sheep showed a closely overlapping ROH island at 34.25–38.24 Mb containing *SPP1*, *PKD2*, and *LCORL* (22, 31). Yurchenko et al. (43) further identified selection signatures involving *LAP3*, *NCAPG*, and *LCORL* in Russian sheep breeds and highlighted these genes in relation to growth and feed-intake traits. Rochus et al. (6) likewise identified *ABCG2* and *LCORL*/*NCAPG* among recurrent selection hotspots in sheep and proposed an introgression-based historical scenario for this region, emphasizing its complex selection history across breeds. At whole-genome sequence resolution, Naval-Sanchez et al. (44) identified a selective sweep spanning the *LCORL* promoter in domestic sheep, whereas mouflon showed a distinct sweep downstream of *NCAPG*, providing further evidence for repeated selection at this locus during domestication and breed formation. Collectively, these studies indicate that the broader OAR6 interval harboring the Ramlıç signal has repeatedly emerged as a target of selection across geographically and phenotypically diverse sheep populations.

The biological relevance of the Ramlıç interval is further reinforced by phenotype-based evidence. In Australian Merino sheep, Al-Mamun et al. (45) identified a major body-weight QTL between 36.15 and 38.56 Mb, directly overlapping the present signal, with *LAP3*, *NCAPG*, and *LCORL* highlighted as positional candidates. The same broader interval also contains *ABCG2*, *SPP1*, *MEPE*, *IBSP*, *FAM184B*, and other genes previously considered relevant to production traits. Variants in *SPP1* were associated with birth and yearling weight, whereas variants in *LAP3* were associated with birth and early postnatal body weights in sheep (46). Beyond growth, Gutiérrez-Gil et al. (47) identified OAR6 among core candidate convergence regions associated with dairy-related selection and highlighted *ABCG2* and *SPP1* as strong positional candidates. Together with the growth-related evidence, this suggests that selection within this broader locus may have involved multiple economically important phenotypes rather than a single production trait. Consistent with this interpretation, whole-genome selection scans in South African Mutton Merino identified *LCORL*, *NCAPG*, *IBSP*, and *FAM184B* among genes supported by at least two selection-signature methods and previously related to growth, carcass traits, and meat quality (48). Moreover, a multi-breed GWAS of mature body size independently identified the *NCAPG*/*LCORL* locus, reinforcing its recurrent association with body size and conformation in sheep (49).

Importantly, the relevance of this region is also supported by previous GWAS conducted within the same Afyon Kocatepe University institutional breeding flock. In Ramlıç-cross lambs, Demirtaş (50) identified an intronic FAM184B SNP at 37.14 Mb associated with 90-day chest width, together with several *GRID2* variants in the broader OAR6 region associated with body measurements. The thesis itself concerned genomic regions associated with growth and ultrasonic carcass traits in Ramlıç-cross lambs. In a subsequent thematic GWAS conducted in the same institutional flock, an intronic SNP in *MEPE* was chromosome-wide significant for birth weight; the project used OvineSNP50K genotypes and specifically identified *MEPE* among candidate genes associated with growth-related traits (51). Although these studies involved different cohorts and phenotypes, their positional agreement within the same breeding population provides population-specific support complementary to the independent signals reported in other sheep breeds. Taken together, the convergence of selection signals across independent sheep populations and genomic approaches, their repeated association with economically relevant phenotypes, and the population-specific GWAS evidence from the same breeding flock provide strong support for OAR6:36.5–38.0 Mb as a major candidate region shaped by selection in Ramlıç sheep.

Beyond the major OAR6 signal, the OAR2 region containing *TYRP1* was particularly noteworthy. *TYRP1* is a well-established determinant of pigmentation in sheep: a functional variant was strongly associated with coat colour in Soay sheep, while independent studies identified *TYRP1* haplotypes and loss-of-function alleles associated with skin, fiber, and coat pigmentation in other sheep populations (52–54). Thus, the concordant ROH–iHS signal encompassing *TYRP1* may be consistent with historical selection related to pigmentation, although the underlying selective phenotype cannot be determined from the present data. The OAR11 overlap containing *TIMP2* and *SOCS3* is also noteworthy, as both genes were recently identified by integrated GWAS and transcriptomic analyses as candidates for perirenal fat deposition in Hu sheep (55). Other concordant regions currently lack comparable functional or phenotype-specific evidence and should therefore be regarded as exploratory candidates requiring further validation. Although these findings provide biologically plausible candidates beyond the major OAR6 signal, the remaining concordant regions currently lack comparable functional or phenotype-specific evidence and should therefore be regarded as exploratory candidates requiring further validation.

## 5 Conclusion

This study characterized genome-wide autozygosity and putative selection signatures in Ramlıç sheep using complementary ROH- and iHS-based approaches. The population showed a mean FROH of 4.57%, with considerable variation among individuals, while short-to-intermediate ROH segments predominated. The joint evaluation of ROH islands and iHS candidate regions identified 15 concordant genomic intervals, providing a focused set of regions potentially influenced by selection. The most prominent concordance was observed on OAR6, particularly at 36.5–38.0 Mb, where the ROH island with the highest maximum ROH incidence coincided with a cluster of extreme |iHS| signals and encompassed several genes previously associated with economically important traits in sheep, including *ABCG2, SPP1, MEPE, LAP3,* and *NCAPG*. Additional concordant signals on other autosomes suggest that the genomic footprints identified in Ramlıç are not restricted to OAR6. Overall, these findings provide new insights into genomic autozygosity and potential selection history in Ramlıç sheep and identify candidate regions that warrant further investigation using larger populations, higher-resolution genomic data, and functional validation.

## Data Availability

The data supporting the findings of this study are available from the corresponding author upon reasonable request. Genotype data are not publicly available because they are subject to institutional data management policies and ongoing research activities. Requests to access these datasets should be directed to Orhan Eser, vetorhan95@gmail.com.
