# Supplementary material for: Genome-wide selection signatures in Ramlıç sheep identified by complementary ROH and iHS analyses
Source: Front Vet Sci. 2026 Oct 13;13:2009151. doi: 10.3389/fvets.2026.2009151 (PMC13712115; doi:10.3389/fvets.2026.2009151)
Supplement: Supplementary file 1 [file Table_1.DOCX]

**Supplementary Table S1. Complete list of runs of homozygosity (ROH) islands identified in Ramlıç sheep.**

| **Island** | **OAR** | **Start (Mb)** | **End (Mb)** | **Length (Mb)** | **SNPs** | **Max. ROH (n/96)** | **Max. frequency (%)** | **Peak SNP** | **Peak position (Mb)** |
| --- | --- | --- | --- | --- | --- | --- | --- | --- | --- |
| 23 | 6 | 30.332 | 40.001 | 9.669 | 159 | 25 | 26.04 | OAR6_40780034.1 | 36.573 |
| 24 | 6 | 43.754 | 49.733 | 5.979 | 114 | 19 | 19.79 | OAR6_51969295.1 | 46.891 |
| 10 | 2 | 21.690 | 24.222 | 2.532 | 55 | 16 | 16.67 | s72217.1 | 22.763 |
| 63 | 19 | 20.155 | 25.753 | 5.598 | 94 | 15 | 15.62 | OAR19_22671203.1 | 21.576 |
| 16 | 3 | 28.150 | 30.986 | 2.835 | 57 | 15 | 15.62 | OAR3_32039232.1 | 29.886 |
| 4 | 1 | 74.387 | 78.138 | 3.752 | 84 | 13 | 13.54 | OAR1_79590680.1 | 74.387 |
| 12 | 2 | 80.033 | 83.820 | 3.787 | 79 | 13 | 13.54 | OAR2_86146743.1 | 80.984 |
| 20 | 3 | 130.889 | 133.443 | 2.553 | 48 | 13 | 13.54 | OAR3_140679608.1 | 131.721 |
| 41 | 11 | 51.552 | 53.676 | 2.124 | 37 | 13 | 13.54 | OAR11_56856134.1 | 53.128 |
| 18 | 3 | 105.894 | 111.747 | 5.853 | 105 | 12 | 12.50 | OAR3_112822823.1 | 106.053 |
| 21 | 3 | 184.444 | 187.634 | 3.190 | 58 | 12 | 12.50 | OAR3_200054679.1 | 185.804 |
| 1 | 1 | 28.392 | 30.493 | 2.101 | 40 | 12 | 12.50 | s32583.1 | 29.284 |
| 53 | 16 | 54.870 | 56.088 | 1.218 | 25 | 12 | 12.50 | OAR16_60075688.1 | 55.120 |
| 31 | 9 | 21.946 | 24.527 | 2.581 | 60 | 11 | 11.46 | OAR9_23529045.1 | 22.624 |
| 57 | 17 | 49.864 | 52.959 | 3.096 | 50 | 11 | 11.46 | s10425.1 | 50.179 |
| 66 | 24 | 32.268 | 35.559 | 3.291 | 47 | 11 | 11.46 | s03477.1 | 34.461 |
| 28 | 7 | 51.221 | 53.207 | 1.986 | 45 | 11 | 11.46 | OAR7_56597763.1 | 51.221 |
| 62 | 18 | 30.478 | 32.888 | 2.410 | 44 | 11 | 11.46 | s19976.1 | 30.478 |
| 49 | 15 | 52.773 | 55.204 | 2.431 | 43 | 11 | 11.46 | s04508.1 | 52.945 |
| 50 | 16 | 14.767 | 16.868 | 2.101 | 43 | 11 | 11.46 | OAR16_17147237.1 | 15.515 |
| 30 | 8 | 21.985 | 24.192 | 2.207 | 40 | 11 | 11.46 | OAR8_24432218.1 | 21.985 |
| 48 | 15 | 38.219 | 39.976 | 1.757 | 38 | 11 | 11.46 | OAR5_11359709.1 | 38.322 |
| 6 | 1 | 117.776 | 120.476 | 2.700 | 37 | 11 | 11.46 | OAR1_127060133.1 | 117.776 |
| 33 | 9 | 26.266 | 28.456 | 2.189 | 36 | 11 | 11.46 | OAR9_27249506_X.1 | 26.266 |
| 11 | 2 | 69.742 | 70.665 | 0.923 | 15 | 11 | 11.46 | OAR2_74565025.1 | 69.891 |
| 60 | 18 | 3.074 | 4.109 | 1.036 | 14 | 11 | 11.46 | OAR18_2613628.1 | 3.074 |
| 40 | 11 | 21.549 | 22.074 | 0.526 | 14 | 11 | 11.46 | s59074.1 | 22.074 |
| 15 | 2 | 234.212 | 234.761 | 0.549 | 13 | 11 | 11.46 | OAR2_247800434.1 | 234.656 |
| 45 | 14 | 37.259 | 37.862 | 0.603 | 12 | 11 | 11.46 | OAR14_38797310.1 | 37.296 |
| 38 | 11 | 19.398 | 19.943 | 0.545 | 12 | 11 | 11.46 | OAR11_19810690.1 | 19.408 |
| 26 | 6 | 86.368 | 86.871 | 0.504 | 10 | 11 | 11.46 | s59224.1 | 86.540 |
| 47 | 15 | 37.656 | 37.968 | 0.312 | 7 | 11 | 11.46 | OAR15_39506759.1 | 37.656 |
| 44 | 14 | 36.602 | 36.635 | 0.032 | 2 | 11 | 11.46 | OAR14_38093758.1 | 36.635 |
| 32 | 9 | 25.683 | 25.683 | 0.000 | 1 | 11 | 11.46 | s43788.1 | 25.683 |
| 7 | 1 | 235.315 | 236.525 | 1.210 | 29 | 10 | 10.42 | OAR1_254059966.1 | 235.315 |
| 65 | 20 | 43.994 | 44.834 | 0.840 | 20 | 10 | 10.42 | OAR20_47837199.1 | 43.994 |
| 46 | 15 | 36.386 | 37.497 | 1.111 | 18 | 10 | 10.42 | s19222.1 | 36.386 |
| 36 | 9 | 42.012 | 42.718 | 0.706 | 16 | 10 | 10.42 | OAR9_44167764.1 | 42.012 |
| 14 | 2 | 229.561 | 230.359 | 0.798 | 15 | 10 | 10.42 | OAR2_242398018.1 | 229.561 |
| 9 | 1 | 244.170 | 244.942 | 0.772 | 15 | 10 | 10.42 | s64605.1 | 244.170 |
| 64 | 19 | 47.126 | 47.848 | 0.722 | 15 | 10 | 10.42 | s40008.1 | 47.126 |
| 37 | 11 | 3.646 | 4.339 | 0.694 | 13 | 10 | 10.42 | OAR11_3002948.1 | 3.646 |
| 27 | 7 | 49.762 | 50.580 | 0.819 | 12 | 10 | 10.42 | s10805.1 | 49.762 |
| 54 | 16 | 60.407 | 60.909 | 0.502 | 11 | 10 | 10.42 | OAR16_65917082.1 | 60.407 |
| 34 | 9 | 28.567 | 28.963 | 0.396 | 9 | 10 | 10.42 | OAR9_29869169.1 | 28.567 |
| 22 | 4 | 60.257 | 60.691 | 0.434 | 8 | 10 | 10.42 | OAR4_63706459.1 | 60.257 |
| 42 | 11 | 54.137 | 54.510 | 0.372 | 8 | 10 | 10.42 | OAR11_57956809.1 | 54.137 |
| 56 | 16 | 68.258 | 68.564 | 0.306 | 8 | 10 | 10.42 | OAR16_74158379.1 | 68.258 |
| 35 | 9 | 32.549 | 32.816 | 0.266 | 6 | 10 | 10.42 | s62043.1 | 32.549 |
| 2 | 1 | 64.888 | 65.156 | 0.268 | 5 | 10 | 10.42 | OAR1_68635821.1 | 64.888 |
| 58 | 17 | 53.090 | 53.294 | 0.204 | 5 | 10 | 10.42 | s30684.1 | 53.090 |
| 13 | 2 | 152.092 | 152.216 | 0.124 | 5 | 10 | 10.42 | s65249.1 | 152.092 |
| 3 | 1 | 66.070 | 66.312 | 0.242 | 4 | 10 | 10.42 | OAR1_70611692.1 | 66.070 |
| 17 | 3 | 79.592 | 79.711 | 0.119 | 4 | 10 | 10.42 | OAR3_84154568.1 | 79.592 |
| 19 | 3 | 124.728 | 125.040 | 0.312 | 3 | 10 | 10.42 | OAR3_132998265.1 | 124.728 |
| 55 | 16 | 65.317 | 65.430 | 0.113 | 3 | 10 | 10.42 | OAR16_71075325_X.1 | 65.317 |
| 29 | 7 | 60.674 | 60.762 | 0.087 | 3 | 10 | 10.42 | OAR7_66581251.1 | 60.674 |
| 43 | 12 | 38.475 | 38.585 | 0.110 | 2 | 10 | 10.42 | OAR12_42878639.1 | 38.475 |
| 5 | 1 | 78.916 | 79.001 | 0.085 | 2 | 10 | 10.42 | OAR1_84220386.1 | 78.916 |
| 39 | 11 | 20.456 | 20.525 | 0.068 | 2 | 10 | 10.42 | s74228.1 | 20.456 |
| 61 | 18 | 29.724 | 29.789 | 0.066 | 2 | 10 | 10.42 | s61032.1 | 29.724 |
| 51 | 16 | 32.922 | 32.946 | 0.023 | 2 | 10 | 10.42 | OAR16_35801820.1 | 32.922 |
| 8 | 1 | 236.639 | 236.639 | 0.000 | 1 | 10 | 10.42 | s08489.1 | 236.639 |
| 25 | 6 | 49.852 | 49.852 | 0.000 | 1 | 10 | 10.42 | OAR6_55140323.1 | 49.852 |
| 52 | 16 | 42.884 | 42.884 | 0.000 | 1 | 10 | 10.42 | OAR16_46789766.1 | 42.884 |
| 59 | 17 | 57.911 | 57.911 | 0.000 | 1 | 10 | 10.42 | OAR17_63349788.1 | 57.911 |

The table presents the complete set of 66 ROH islands identified across 96 genotyped Ramlıç sheep. ROH islands were defined from consecutive SNPs showing elevated occurrence within ROH. Max. ROH (n/96) indicates the highest number of individuals in which a SNP within the island occurred in an ROH, and maximum frequency (%) represents the corresponding proportion of the 96 animals. Peak SNP denotes the SNP with the highest ROH frequency within each island. OAR, Ovis aries autosome; ROH, runs of homozygosity; SNP, single nucleotide polymorphism; Mb, megabase.

**Supplementary Table S2. Genomic regions identified as candidate selection signatures based on iHS analysis in Ramlıç sheep.**

| **OAR** | **Genomic region (Mb)** | **SNPs in window** | **Top 1% SNPs** | **Top 1% (%)** | **Max \|iHS\|** |
| --- | --- | --- | --- | --- | --- |
| 1 | 28.5–29.0 | 9 | 3 | 33.33 | 3.834 |
| 2 | 21.5–22.0 | 10 | 3 | 30.00 | 3.667 |
| 2 | 67.0–67.5 | 11 | 4 | 36.36 | 3.783 |
| 2 | 80.5–81.0 | 15 | 5 | 33.33 | 3.282 |
| 2 | 114.0–114.5 | 6 | 5 | 83.33 | 4.376 |
| 2 | 114.5–115.0 | 10 | 7 | 70.00 | 4.564 |
| 2 | 203.0–203.5 | 12 | 4 | 33.33 | 3.114 |
| 2 | 215.0–215.5 | 9 | 3 | 33.33 | 3.229 |
| 2 | 215.5–216.0 | 8 | 4 | 50.00 | 3.859 |
| 3 | 38.0–38.5 | 10 | 3 | 30.00 | 3.541 |
| 5 | 6.0–6.5 | 9 | 6 | 66.67 | 3.586 |
| 6 | 32.0–32.5 | 8 | 8 | 100.00 | 4.458 |
| 6 | 32.5–33.0 | 5 | 5 | 100.00 | 5.492 |
| 6 | 34.5–35.0 | 12 | 5 | 41.67 | 3.434 |
| 6 | 36.5–37.0 | 6 | 6 | 100.00 | 6.011 |
| 6 | 37.0–37.5 | 7 | 7 | 100.00 | 5.697 |
| 6 | 37.5–38.0 | 9 | 8 | 88.89 | 4.324 |
| 6 | 44.5–45.0 | 6 | 3 | 50.00 | 3.476 |
| 7 | 21.0–21.5 | 10 | 8 | 80.00 | 5.498 |
| 8 | 52.0–52.5 | 12 | 3 | 25.00 | 3.457 |
| 9 | 50.0–50.5 | 7 | 7 | 100.00 | 3.902 |
| 9 | 69.0–69.5 | 8 | 6 | 75.00 | 4.010 |
| 9 | 69.5–70.0 | 9 | 6 | 66.67 | 3.696 |
| 10 | 36.5–37.0 | 13 | 4 | 30.77 | 3.305 |
| 11 | 21.5–22.0 | 11 | 4 | 36.36 | 3.683 |
| 11 | 22.0–22.5 | 9 | 7 | 77.78 | 4.099 |
| 11 | 22.5–23.0 | 9 | 3 | 33.33 | 3.922 |
| 11 | 52.0–52.5 | 8 | 3 | 37.50 | 4.456 |
| 11 | 52.5–53.0 | 9 | 7 | 77.78 | 4.974 |
| 11 | 53.0–53.5 | 7 | 3 | 42.86 | 3.733 |
| 11 | 56.0–56.5 | 7 | 6 | 85.71 | 4.166 |
| 11 | 56.5–57.0 | 5 | 3 | 60.00 | 3.438 |
| 11 | 60.5–61.0 | 9 | 3 | 33.33 | 4.149 |
| 12 | 5.0–5.5 | 6 | 4 | 66.67 | 4.445 |
| 13 | 50.0–50.5 | 6 | 3 | 50.00 | 3.424 |
| 14 | 13.5–14.0 | 8 | 3 | 37.50 | 3.179 |
| 14 | 16.0–16.5 | 11 | 5 | 45.45 | 3.333 |
| 16 | 13.0–13.5 | 11 | 3 | 27.27 | 3.449 |
| 19 | 53.0–53.5 | 10 | 4 | 40.00 | 3.302 |
| 21 | 25.5–26.0 | 10 | 4 | 40.00 | 3.667 |
| 21 | 39.0–39.5 | 5 | 4 | 80.00 | 4.444 |
| 21 | 39.5–40.0 | 11 | 6 | 54.55 | 3.611 |
| 23 | 26.0–26.5 | 6 | 3 | 50.00 | 3.686 |
| 23 | 26.5–27.0 | 10 | 4 | 40.00 | 4.917 |
| 24 | 10.5–11.0 | 6 | 6 | 100.00 | 4.394 |
| 25 | 6.5–7.0 | 9 | 7 | 77.78 | 4.078 |
| 26 | 24.0–24.5 | 7 | 5 | 71.43 | 3.810 |
| 26 | 24.5–25.0 | 12 | 3 | 25.00 | 3.756 |

Candidate regions were identified using non-overlapping 500-kb windows. Windows containing at least three SNPs exceeding the empirical Top 1% threshold (|iHS| ≥ 2.7232) were considered candidate selection signatures. A total of 48 candidate windows distributed across 20 autosomes met this criterion. OAR, Ovis aries autosome; SNP, single nucleotide polymorphism; iHS, integrated haplotype score.
